# Incorporating dietary fiber from fruit and vegetable waste in meat products: a systematic approach for sustainable meat processing and improving the functional, nutritional and health attributes

**DOI:** 10.7717/peerj.14977

**Published:** 2023-03-01

**Authors:** Abdul Haque, Saghir Ahmad, Z. R. A. A. Azad, Mohd Adnan, Syed Amir Ashraf

**Affiliations:** 1 Department of Post-Harvest Engineering and Technology, Faculty of Agricultural Sciences, Aligarh Muslim University, Aligarh, Uttar Pardesh, India; 2 Department of Biology, College of Science, University of Ha’il, Ha’il, Saudi Arabia; 3 Department of Clinical Nutrition, College of Applied Medical Sciences, University of Ha’il, Ha’il, Saudi Arabia

**Keywords:** Meat products, Fruit waste, Vegetable waste, Food by-product, Dietary fiber, Nutraceuticals, Shelf-life, Metabolic disorders, Gastrointestinal disorders

## Abstract

**Background:**

Every year, the food business produces a sizeable amount of waste, including the portions of fruits and vegetables that are inedible, and those that have reached a stage where they are no longer suitable for human consumption. These by-products comprise of components such as natural antioxidants (polyphenols, carotenoid *etc*.), dietary fiber, and other trace elements, which can provide functionality to food. Due to changing lifestyles, there is an increased demand for ready-to-eat products like sausages, salami, and meat patties. In this line, meat products like buffalo meat sausages and patties are also gaining the interest of consumers because of their rich taste. Meat, however, has a high percentage of fat and is totally deprived of dietary fiber, which poses severe health problems like cardiovascular (CV) and gastrointestinal diseases. The health-conscious consumer is becoming increasingly aware of the importance of balancing flavor and nutrition. Therefore, to overcome this problem, several fruit and vegetable wastes from their respective industries can be successfully incorporated into meat products that provide dietary fiber and play the role of natural antioxidants; this will slow down lipid oxidation and increase the shelf-life of meat products.

**Methodology:**

Extensive literature searches have been performed using various scientific search engines. We collected relevant and informative data from subject-specific and recent literature on sustainable food processing of wasted food products. We also looked into the various applications of waste fruit and vegetable products, including cereals, when they are incorporated into meat and meat products. All relevant searches meeting the criteria were included in this review, and exclusion criteria were also set.

**Results:**

The pomace and peels of fruits like grapes, pomegranates, cauliflower, sweet lime, and other citrus are some of the most commonly used fruit and vegetable by-products. These vegetable by-products help inhibit oxidation (of both lipids and proteins) and the growth of pathogenic and spoilage bacteria, all without altering the consumer’s acceptability of the product on a sensory level. When included in meat products, these by-products have the potential to improve the overall product quality and lengthen its shelf-life under certain circumstances.

**Conclusion:**

Cost-effective and easily accessible by-products from the fruit and vegetable processing industries can be used in meat products to enhance their quality features (physicochemical, microbial, sensory, and textural aspects) and health benefits. Additionally, this will provides environmental food sustainability by lowering waste disposal and improving the food’s functional efficacy.

## Introduction

Meat is a fundamental component of any balanced diet. Compared to other food sources, meat has a greater protein concentration and a higher degree of bioavailability (Chan, 2004; Biesalski, 2005). In addition, meat is an excellent source of omega-3 fatty acid, cobalamin and iron, containing a high level of all three nutrients (Sharma, Sheehy & Kolonel, 2013). There is a growing trend and popularity among consumers of processed meat products such as sausages, meat patties, kebabs, bologna, meat batters, frankfurters, meatballs, fermented sausage, burgers, *etc*. (Daniel et al., 2011; Ritchie, Rosado & Roser, 2017; Shan et al., 2017). Processed meat products are becoming increasingly popular in the developed world due to rising customer demand for their convenience and perceived high quality in terms of taste, flavor, texture, and nutritional profile (Shan et al., 2017; Yeung & Huang, 2017).

However, the perception of meat fat as high in saturated fatty acids (SFA) has led to the belief that meat, particularly red meat, should be avoided. Also, the lack of dietary fiber (DF) in meat and processed meat is responsible for the unfavorable effects associated with these foods. Many health issues, including hypertension, coronary heart disease, obesity, and cardiovascular illnesses, have been linked to regular meat consumption (Micha, Michas & Mozaffarian, 2012). Specific components in diet have been linked to neurological disorders, which has been supported by scientific research (Gómez-Pinilla, 2008). The trend toward healthier food among consumers has led to an uptick in the market for functional meat products in recent years. As a direct consequence, products made from meat are now incorporating plenty of valuable bioactive components (Rashwan et al., 2020). Incorporating ingredients sourced from other natural sources that have been shown to enhance physiological functions of the body, such as increased immunity and anti-aging, qualifies a food product as a functional food (Chappalwar et al., 2020; Elkhalifa et al., 2021).

There is growing evidence that plant meals, especially whole grains and vegetables, are a great way to get the fiber your body needs (Stephen et al., 2017). DF is a type of carbohydrate found in plants that cannot be broken down by the digestive system and absorption by the body in the small intestine (Soliman, 2019). Furthermore, it’s been noticed that around the worldwide, human beings usually eat less than 20 g of DF daily (Stephen et al., 2017) whereas using the energy guideline of 2,000 kcal/day for women and 2,600 kcal/day for men, the recommended daily dietary fiber intake is 28 g/day for adult women and 36 g/day for adult men (USDA, 2005). Because of this, it is essential to produce a variety of DF containing food items. DF can be incorporated to meat products for various health benefits, since meat does not naturally contain any DF. It is anticipated that the need of meat products will expand dramatically in developing nations, and to some extent, it will also increase in developed nations countries (Future Market Insights Global and Consulting Pvt. Ltd., 2022, www.futuremarketinsights.com). As a result, the various meat products have the potential to be enrich with beneficial compounds, while the concentration of compounds that are disadvantageous can be reduced. Therefore, many components can be added to meat products without changing their fundamental qualities (Bhat, 2011). In addition to the health benefits, which customers enjoy, the DF-enriched meat products have enhanced functional features that make them more desirable to buyers (Aleson-Carbonell et al., 2005). Only a few researchers looked at the feasibility of various components. Still, they were able to come up with a functional food that is both safe and effective due to the addition of phytochemicals (Banerjee et al., 2020; Santhi, Kalaikannan & Natarajan, 2020).

Meat and meat products are highly perishable because of their complex composition and high water content, which promotes the growth and action of bacteria associated with decomposition and causes sensory and nutritional modifications due to lipids and proteins oxidation, among other things (Falowo, Fayemi & Muchenje, 2014; Amaral, Da Silva & Da Silva Lannes, 2018). There are various bioactive compounds such as antioxidant, antimicrobials, and novel processing technologies that have been used to extend the storage life of meat products. Natural compounds or substances and methods that employ few or no thermal reactions have been the focus of much attention (Jayasena & Jo, 2013; Sánchez-Ortega et al., 2014; Hugo & Hugo, 2015; Ur Rahman et al., 2018). Moreover, waste materials obtained from agro-industrial industries have acquired importance in the context of technologies that utilize natural compounds, because they represent a source of underutilized, but value-adding compounds or substances such as polyphenols, DF, antioxidants and terpenes (Mamma & Christakopoulos, 2014). The production of wine, juice, fruit and vegetable products like jam, marmalade, and oleoresins, are just some of the businesses responsible for producing a significant portion of these wastes, among others. It has been observed that the residues from these industries contains polyphenols and fiber (Devi et al., 2009; Quezada & Cherian, 2012). In general, agro-industrial wastes consist of a considerable percentage of inedible parts of fruits. These inedible parts can include seeds, shells, petals, and roots in some instances, and their weight can account for as much as 10 to 30 percent of the fruit’s overall mass. Utilizing these low-cost by-products from the food processing sectors can lead to new forms of indirect revenue and environmental sustainability. The potential pollution caused by discarding vegetable and fruit wastes is mitigated by their use as a source of nutritional fiber and natural antioxidants. As a result, not only does it benefit the environment by reducing the burden of their disposal, but it also aids the economy by reducing waste. Consumers are attracted to meat products that use plant by-products because they enhance the product’s overall quality, functionality, and shelf life (Pintado et al., 2016; Calderón-Oliver & López-Hernández, 2022). This also develops a sign of satisfaction among the non-vegetarian consumers regarding their diet.

Production of food waste materials in food processing industries is huge, and as a result, a valuable source of potential revenues is getting lost, and the cost of disposing these products is also increasing day by day. In addition to economic losses, unused waste material causes serious environmental concerns and possesses a threat for human health hazards. Hence, improved utilization of food waste materials could help to combat the food insecurity as well as provide potential health benefits as food waste possess several important bioactive compounds. Therefore, this review brings up a current update on incorporation of food waste material into meat products and its significance for improving the nutritional as well as functional characteristics. In addition, this review takes along all the possible food waste materials incorporation enriched with bioactive components to advance the efficacy of food product. Furthermore, the presented review will encourage the food scientific communities, food industries and ready to eat food product produces to bring up more sustainable way of producing food product for consumers with better functional characteristics. Subsequently, such innovative and sustainable way of meat fortification will increase the confidence of consumers while eating meat products.

## Survey methodology

Various scientific search engines like Science Direct, PubMed, Scopus, *etc*. were searched and approximate number of articles published in last 20 years were retrieved from different search engines of scientific literature. Keywords/phrases used to search relevant data were meat and meat products, sustainable food processing of wasted food, food and vegetable waste, improving the food functional efficacy, by-products from the fruit and vegetable processing industries, health benefits of dietary fiber *etc*., which reflected only subject-related literature. Exclusion criteria were also set. Studies that do not meet the current inclusion criteria, irrelevant to the topic, abstracts, conference proceedings, editorials and commentary with insufficient data were excluded.

### Nutritional value of meat

The prevalent levels of saturated fat in meat, especially red meat, give it a bad reputation regarding people’s health. Therefore, it is recommended to limit one’s consumption of meat, particularly red meat, in order to reduce an individual’s chance of developing certain disorders and diseases including cardiovascular (CV) diseases (Giromini & Givens, 2022). However, this perspective takes into account the fact that meat is a rich source of several micronutrients, including vitamins and trace minerals that are either absent from meals derived from plants or have a low bioavailability in those foods (Biesalski, 2005). In addition, because it is high in protein and low in carbohydrates, meat has a lesser glycemic index (GI), which is thought to be advantageous in preventing obesity, diabetes, and cancer. Meat is a product that is rich in protein and low in carbohydrates (Biesalski, 2005).

### Protein content of meat

Protein makes up about twenty percent of the average muscle’s composition. Lean meat’s dry matter is primarily made up of proteins, the most abundant component (Biesalski, 2005). Because the human body is unable to produce nine of the amino acids that are found in proteins from other substances, the body must obtain these amino acids from the food it consumes. These amino acids are considered essential or semi-essential. Meat typically has its protein in its protein at relatively high concentrations of the four most important amino acids-sulfur-containing amino acids, lysine, threonine, and tryptophan. It should not come as a surprise that the protein quality of animal proteins, such as those found in meat and milk, tends to be higher than that of plant proteins. When compared to plant proteins, the proteins found in animals are easier to digest. The fact that the majority of plant proteins are wrapped in polysaccharide matrix, inaccessible to proteolytic enzymes, helps to explain this phenomenon to some extent. The protein content of a meal can be improved by the complementing effect of combining plant and animal sources (Colmenero, Ayo & Carballo, 2005).

Proteins included in meat are essential for human nutrition. Since animal proteins must be digested into amino acids or tiny peptides before they can pass through the small intestinal wall and into the bloodstream, their nutritional value is directly related to how easily they can be broken down (Sun, Zhou & Zhao, 2015). Changes in meat protein aggregation and oxidation can affect its digestion by digestive enzymes (Sante-Lhoutellier, Aubry & Gatellier, 2007). Incorporation of plant-based waste in meat products generally does not harm protein digestibility and quality (Lin et al., 2019). Also in another study, Jucara (*Euterpe edulis*) fruit waste extract was able to stabilize protein oxidation in conventional broiler meat (da Silva Frasao et al., 2021). So, incorporating fruit and vegetable in meat products is safer regarding protein digestibility and helps in stabilizing the protein oxidation.

### Fat content of meat

Animal fat is primarily stored in fatty tissue, which can be further subdivided into adipose fat, subcutaneous fat, intermuscular fat, and marbling fat. The fat found within the muscles is known as marbling, and it helps produce a favorable texture (Lawrie & Ledward, 2014). Depending on the animal’s fat excretion and the preparation method, a given piece of meat may include varying amounts of intermuscular and depot fat. Saturated fatty acids (SFA) are commonly thought to make up the bulk of animal fat, whereas over 50% of the fatty acids in meat are unsaturated (Lawrie & Ledward, 2014). Lipids in meat typically comprise less than half saturated fatty acids (beef 50–52%) and as much as 70% unsaturated fatty acids (Valsta, Tapanainen & Männistö, 2005).

The grinding, cooking, and storing steps in the processing of meat products expose lipids to the air, which causes them to oxidize quickly and irreversibly. Meat and meat products lose their desirable flavor and texture because of rancidity, turn brown, and create hazardous substances including malondialdehyde and cholesterol oxidation products due to lipid oxidation (Choe et al., 2014). The addition of various fruit and waste extract may help in retarding lipid oxidation and reducing the fat content of the meat products. In one study, to a more significant extent, persimmon peel extracts prevented lipid oxidation of pork patties while they were being refrigerated (Choe, Kim & Kim, 2017). In another study, inulin from chicory root was able to reduce the fat at a significant level in pork and chicken meatball (Montoya et al., 2022). Thus incorporation of fruit and vegetable waste and their extract in meat products may help in slowing down lipid oxidation and rancidity.

### Vitamin content of meat

Most fat-soluble vitamins are not easily eliminated from the body and are instead retained in the liver and adipose tissues alongside fat. On the other hand, the body tends to keep a far smaller quantity of water-soluble vitamins than it does fat-soluble vitamins. The majority of vitamins found in livestock and human diets are produced either by plants or microorganisms. Some animal cells can synthesize vitamins like vitamin D, niacin, and ascorbate, and convert pro-vitamins to the active form (McDowell, 2008). Additionally, commensal microbes in the digestive tracts of both ruminants and non-ruminants have the potential to operate as a source of some vitamins, including vitamin K and the water-soluble B-complex vitamins (McDowell, 2008). Regarding human nutrition, meat has long been acknowledged as a reliable source of B vitamins (Obeid et al., 2019).

### Mineral content of meat

The zinc and iron in meat are two of its most valuable nutrients. It is well established that meals rich in animal protein produce more excellent zinc absorption than meals rich in wholegrain cereal (Latunde-Dada & Neale, 2007). Selenium (Se) is an essential trace element to maintain good health. Processes like as antioxidative defense mechanism, inflammation lessening, thyroid hormone synthesis, fertility, reproduction, and DNA synthesis rely on this selenoprotein component (Antonyak et al., 2018). The mineral composition of frankfurter-style sausages was drastically altered when buckwheat husk was added. The addition of this non-meat item resulted in a greater concentration of several trace elements, including manganese, calcium, potassium, and magnesium; the first of these increased by nearly six times, and the last, by more than 40 percent (Salejda et al., 2022). The research conducted on the quality of pork loaves with the addition of hemp seeds, de-hulled hemp seeds, hemp protein, and hemp flour confirmed that there was a significant increase in magnesium and manganese after the addition of these non-meat ingredients to a meat product. This increase occurred after the addition of hemp seeds, hemp protein and hemp flour (Zając et al., 2019). Manganese, potassium, and magnesium levels in meat emulsion were all boosted by the addition of house cricket flour in place of lean meat/fat portions (Kim et al., 2017).

Selenium (Se) is an essential trace element to maintain good health. Processes like as antioxidative defense mechanism, inflammation lessening, thyroid hormone synthesis, fertility, reproduction, and DNA synthesis rely on this selenoprotein component (Hariharan & Dharmaraj, 2020).

### Dietary fiber incorporation in meat products and its effect in various metabolic disorders

**Figure 1:**
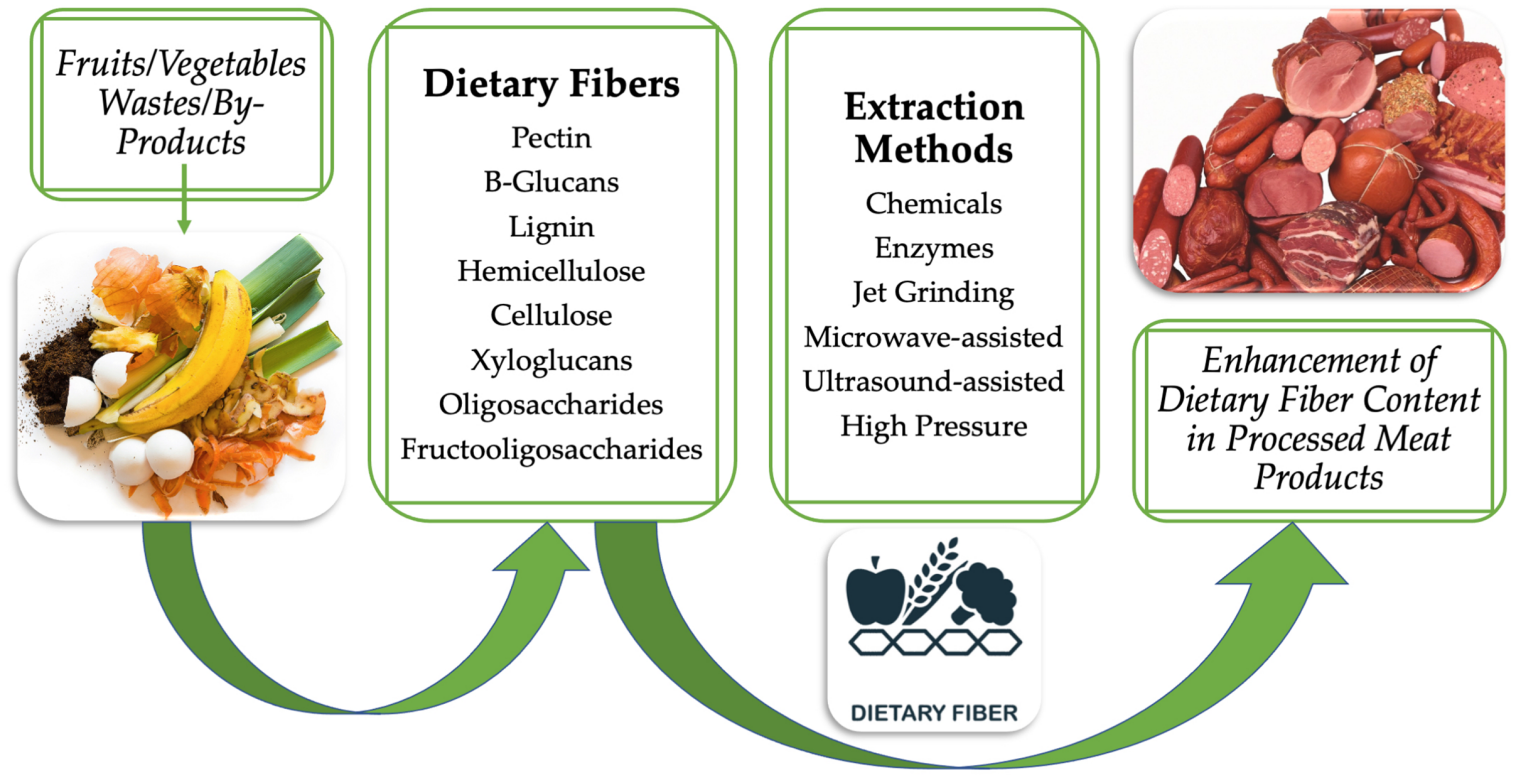
Incorporation of food waste material enriched with dietary fiber to enhance the meat product dietary fiber content.

Dietary fiber is derived from plant sources, especially agro-waste. These dietary fibers are an excellent source of natural antioxidants and may serve to improve the shelf life of meat products when incorporated into them. Antioxidants are an additive used in meat products that could be substituted with natural antioxidants obtained from DF. These substances prevent the oxidation of lipids, proteins, and pigments in foods, extending their shelf life and maintaining the food’s original color, texture, aroma, flavor, and overall quality (Damodaran & Parkin, 2017). Many fruit and vegetable wastes are the attractive source of DF. When these wastes included in different meat products, the DF content was noticed as in one of the studies, Mosambi (sweet lime) peel powder was incorporated in sausages and patties at various levels. After incorporation, 7.33% and 6.24% of total DF were found in sausage and patties, respectively (Younis, Ahmad & Malik, 2021). Similarly, in other study, 3% DF was found by incorporating the upper stem of white cauliflower (7.5%) in beef sausage (Abul-Fadl, 2012). While, in another study, 2.94% DF was found when 40% oyster mushroom was added in chicken sausage (Ahmad et al., 2020) as shown in Fig. 1. The inclusion of dietary fiber in meat products also helps in health management. Waste from fruits and vegetables includes phytic acid (Jayadev, 2017; Ani & Abel, 2018), which acts as a cation exchange component and a mineral chelator for positively charged ions like cadmium, calcium, zinc, and copper (Ekholm et al., 2003). Chelating agents can influence metal toxicity by mobilizing the toxic metal mainly into the urine (Flora & Pachauri, 2010).

Dietary phytochemicals are substances derived from plants that are not considered nutrients but have been connected with a lower risk of developing certain chronic diseases (Liu, 2004). When these phytochemicals are ingested consistently through food, they even have the potential to protect against some malignancies and cardiovascular illnesses (Okarter & Liu, 2010). Soluble dietary fiber, such as barley and oats, protects against heart-related disorders and certain malignancies, and lowers total cholesterol and low-density lipoprotein cholesterol (Pins, 2006). Insoluble dietary fiber, such that found in wheat bran, has been demonstrated to reduce the risk of colon cancer, as well as the risk of developing other cancer, obesity, and gastro-intestinal issues (Stevenson et al., 2012).

### Role of dietary fiber in human health

Roughage-rich diets have been shown to lessen the risk of obesity, CV disease, and several malignancies. DF shows the chances of reduction of several diseases that occurs due to lack of fiber in meat products after incorporating DF from different fruit and vegetable sourced wastes. Foods high in DF have a bulking effect. Over the past few years, an increasing body of research has focused on how to reap the health benefits of DF, mainly by adding fiber to a diet that is otherwise deficient in recent diet due to highly processed food product. DF acts as a laxative, reduces blood cholesterol, and provides antioxidant activity; these are just three of the many benefits that have been tallied during the last half-century. Some DF can’t do what they are credited with doing, while others can’t do it as well or as often.

### Dietary fiber lowers cholesterol and prevents cardiovascular diseases

Because of its ability to lower blood cholesterol and triglyceride levels, DF has gained attention as a potential nutraceutical agent for preventing CV diseases (McRae, 2017). They found that β-glucan or psyllium fibers reduces significantly total serum and LDL cholesterol levels. Diets high in dietary fiber could dramatically lower total cholesterol, on consuming fruits and vegetables in abundance (Soliman, 2019). A few essential mechanisms include the inhibition of lipoprotein formation and cholesterol synthesis, increased insulin sensitivity due to delayed macronutrient absorption (Nweze, Nebechukwu & Bawa, 2021). As a result, consuming DF plays a significant role in reducing the potential of CV diseases.

### Dietary fiber and prevention of gastrointestinal diseases

It is widely known that increasing one’s consumption of fiber results in decreased transit periods, increased feces volume, and enhanced fluid concentration (Soliman, 2019). Compared to the other types of fibers typically taken in through diet, cellulose tends to produce a larger volume of feces, which in turn decreases the transit period (Wichert et al., 2002). It has been demonstrated that DF can absorb various mutagens like Trp-P-1, Trp-P-2, AαC and MeAαC (processed induced mutagenic heterocyclic amines), hence decreasing the amount of time the colon is exposed to these substances (Raman et al., 2013). In short, DF reduces transit time, dilutes and binds toxic colon chemicals, and reduces mucosal exposure to many of these substances. Dietary fiber’s role against colon cancer has been outlined in detail. Anti-carcinogenic activity can be achieved in two ways: (a) by decreasing the synthesis of carcinogenic compounds in the colon and (b) by increasing fecal volume, which decreases the contact of cancer risk agents present in feces with intestinal mucosa (Sharma, Yadav & Ritika, 2008). DF helps lower cancer risk, especially for colorectal cancer, which begins in the large intestine, by promoting fermentation that produces short-chain fatty acids (Xiong et al., 2022).

### Role of dietary fiber in satiety regulation and weight control

Due to the intake of DF in food, the bulk is created, which gives a full stomach feeling and stops a person from eating further for a longer time. Satiety regulation and gastrointestinal emptying are aided by the hormones produced in the gut, which are increased by DF (Costabile et al., 2018). Researchers from all around the world have looked at the weight-control benefits of DF (Puupponen-Pimiä et al., 2002). A total weight loss of 2.494 kg was found in a meta-analysis of studies involving different fiber types (Thompson et al., 2017). Another meta-analysis found that using chitosan supplements decreased nearly 1.814 pounds in total body weight loss (Mhurchu et al., 2005).

### Dietary fiber in the prevention of constipation

Water-insoluble, non-fermentable fibers immediately increase the luminal size, which in turn results in shorter gut transit time, which promotes laxation. On the other hand, water-soluble fibers have a high water-holding capacity, which results in bulky, soft stools that are easier to pass (Slavin, 2013). Two different meta-analyses indicate that fiber supplementation significantly increases stool frequency compared to placebo (Yang et al., 2012; Christodoulides et al., 2016). The risk of getting hemorrhoids may be reduced by taking a fiber supplement; if doing so reduces, the symptoms of constipation and the straining that accompany it.

### Dietary fiber in the prevention of inflammatory bowel disease

There was a 56% decrease in the incidence of Crohn’s disease and a 20% decrease in the incidence of ulcerative colitis, when comparing the highest and lowest categories of DF intake. However, the Crohn’s disease meta-analysis revealed substantial heterogeneity. The incidence of Crohn’s disease was observed to reduce by 13% for every 10 g/d increase in dietary fiber, suggesting a linear dose-response association between the two (Liu et al., 2015). The short-chain fatty acid component of fermentable fiber, butyrate, has been hypothesized to have an anti-inflammatory impact *via* downregulating the transcription factor NF-kB. According to a meta-analysis supporting this anti-inflammatory effect, supplementing with DF led to a small, but statistically significant decrease in C-reactive protein levels in people who were overweight or obese (Jiao et al., 2015).

### Effect of dietary fiber incorporation on physico-chemical properties of meat products

Fiber addition to meat products has changed their overall composition, which has led to the development of new fiber sources and opened up exciting prospects for their application across several industries. The use of dried pumpkin pulp reduced the patties’ moisture content and raised their ash content. Raw and cooked patties both had a higher pH after incorporation. The capacity to retain moisture improved, as the amount of dry pumpkin pulp added rose. The cooking yield and diameter change, when pumpkin pulp was added were not significantly different (Serdaroğlu et al., 2018). This increase in ash content of meat product was probably due to higher mineral content of pumpkin matrix. As meat ash content rises, it could help end specific mineral deficiency. Trace minerals (iron, zinc, selenium, iodine, copper, chromium, manganese and molybdenum) perform vital functions within the body including thyroid metabolism, antioxidant activity and immune function (Vural et al., 2020). For example, zinc (Zn) is the second most abundant trace element in human, which can’t be stored in the body, thus regular dietary intake is required Zn microelement is very essential for male fertility. It could be considered as a nutrient marker with many potentials in prevention, diagnosis, and treatment of male infertility (Fallah, Mohammad-Hasani & Colagar, 2018). In one study, protein content, mineral content, and crude fiber content were increased by the incorporation of white cauliflower by-product flour in beef sausage; pH was found to be increased by the incorporation of flour made from the upper stem of white cauliflower. The cooking yield was also increased gradually with the incorporation of white cauliflower stem powder, because of its water yielding property (Abul-Fadl, 2012). The quantity of protein and fat in emulsified pork meatballs reduced as rice bran was added. In contrast, the amount of carbohydrates in the meatballs grew dramatically as rice bran was added (Choi et al., 2011). The protein level of frankfurter beef sausages was raised by one percentage point, and the ash content was raised dramatically when 7% of the residue was added. The benefits of increased ash content has already been discussed in previous example. Furthermore, overall lipid levels dropped, and this reduction in lipid provided longer shelf life by cutting down the chance of lipid oxidation. The question of what sort of quality this residue adds to the protein remains unanswered (Savadkoohi et al., 2014). A few other examples are showing the changes in different characteristics of meat products by incorporating various plant-based materials, especially waste, have been listed in Table 1.

**Table 1:** Change in quality characteristics of different meat products as influenced by various incorporations.

Product	Incorporation	Characteristics before incorporationat 0 day of study	Characteristics after incorporationat 0 day of study	References
Sausage	Pomegranate peel (3%)	Moisture–61.89% Protein–15.85% Ash–2.82% pH–7.12	Moisture–58.82% Protein–16.32% Ash–3.09% pH–7.15	El-Nashi et al. (2015)
Beef sausage	White cauliflower upperstem flour (7.5%)	Moisture–60.81% Fat–37.75% Fiber–0.92%	Moisture–63.40% Fat–28.40% Fiber–3%	Abul-Fadl (2012)
Frankfurter	Buckwheat by-product (3%)	Storage loss–1.93% Production yield–85.8% Gumminess–18.2 N Chewiness–12.2 Nm	Storage loss–1.36% Production yield–85.5% Gumminess–16.9 N Chewiness–11.5 Nm	Salejda et al. (2022)
Beef patty	Dried pumpkin pulp and seed (5%)	Moisture–59.71% Ash–2.76% pH–5.89 WHC–75%	Moisture–55.83% Ash–2.9% pH–5.92 WHC–79.8%	Serdaroğlu et al. (2018)
Pork chorizo	Oregano essential oil (0.1%)	Shear force–244.35 g Chewiness–180.2 g-mm pH–5.34 Browning index–97.16	Shear force–216.99 g Chewiness–95.43 g-mm pH–5.27 Browning index–103.42	Perales-Jasso et al. (2018)

### Effect of dietary fiber incorporation on functional properties of meat products

Many fruits and vegetable waste acts as DF. This waste, at the same time, contains such compounds, which show antioxidant properties. In addition to protecting cells from damage caused by free radicals, chemicals like polyphenols can also treat diseases and their symptoms by inhibiting inflammatory responses and halting the progression of infection (Shay et al., 2015). As a result, incorporating such substances into meat products may improve their functionality and, thus, their healthfulness. The remnants of fresh dates are the source of polyphenols; when they were included in the formulation of bologna sausages (15% of the total), the finished product had a polyphenol level of 1.02% (Sánchez-Zapata et al., 2011). This finding suggests that adding extracts rich in polyphenolic chemicals to meat products can serve as an antioxidant and provide health benefits to the end user. Lycopene, a carotenoid present in 80-90% of ripe tomatoes, has been linked to various health benefits, including a reduced risk of prostate cancer and CV disease (Friedman, 2013). After 21 days in storage, 0.58 mg of lycopene per 100 g of product was discovered in concentrations of up to 1.2% of the tomato peel in sausage. Lycopene was detected in beef burgers cooked at 180 °C for 2 min (Luisa García, Calvo & Dolores Selgas, 2009). Furthermore, the leftovers from tomatoes can be a source of amino acids and trace elements. By incorporating just 7% of the residue, the protein level was raised by 1%, while also boosting the ash content from 2.18% to 2.45% in frankfurter beef sausages. The percentage of total lipids dropped from 20.07 to 19.4 as a result (Savadkoohi et al., 2014).

Prebiotics are a potential health benefit of fruit and vegetable waste. Prebiotics are elements that can survive stomach acid, mammalian enzymatic hydrolysis, absorption in GI tract; they are fermentable by intestinal flora and hence foster the expansion of beneficial bacteria like probiotics (Gibson et al., 2004). Prebiotics come in many forms, but some common ones include cellulose and fiber. Fiber from nopal flour (2%) and pineapple peel flour (3%) added to cooked sausages helped inoculated thermos-tolerant (probiotic) lactic acid bacteria thrive over 20 days in storage (Díaz-Vela, Totosaus & Pérez-Chabela, 2015). It is important to note that the amount of bacteria in a formulation such as this one, which contains both a probiotic and a prebiotic, needs to be closely controlled because the bacteria have the potential to degrade the overall quality of the product. It has also been observed in the above-discussed case that the inclusion of agro-industrial waste raises the mineral content of the meat products, which could lead to a rise in mineral consumption and help meet dietary guidelines.

### Effect of dietary fiber incorporation on sensory, textural, and color characteristics of meat products

Important sensory requirements for customer acceptance of meat products include aroma, flavor, color, appearance, tenderness, and juiciness. Adding pea cotyledon fiber to low-fat beef patties (10% and 14% fat) improved tenderness without adversely affecting the juiciness or intensity of the meat flavor (Luisa García, Cáceres & Dolores Selgas, 2006). While adding lemon albedo to fermented sausages did not change the sausages’ odor, granularity, or salty or acidic taste, it did make them juicier. On the other hand, incorporating fresh lemon albedo enhanced the red color’s perceptual impact (Aleson-Carbonell et al., 2003). Tomato peel extract added to ground beef at concentrations of 1.5%, 3%, and 4.5%resulted in highly acceptable grades for the final product; however, the orange appearance and flavor linked with a 6% addition reduced the acceptance rating due to high concentration of this ingredient (Savadkoohi et al., 2014).

The acceptability and desire to purchase a food product and the quality are all reflected in its aesthetic (texture and color), especially for meat products. Concentrations of fiber from citrus in meat products below 1.5% (0.5% and 1%) increased hardness (Fernandez-Gines et al., 2003). In comparison, concentrations of fiber from citrus in meat products at 2% reduced it, possibly as a result of the effect on the free water retention capacity; the final properties of the texture can be modified by the amount of fiber incorporation in the meat product (Fernandez-Gines et al., 2003). When raw albedo was added to fermented sausages, the sausages had lower chewiness values compared to the control, but when cooked albedo was added, the chewiness was greater than the control. Therefore, the addition of the type of lemon albedo (raw or cooked) in fermented sausages was capable of modifying the chewiness of the product. So to remove the bad effect associated with albedo incorporation in sausage was removed by cooking albedo prior to its incorporation in meat product. Similarly, in another study, pretreatment of sodium chloride was given to peel to remove/reduce the bitterness of the sausage arising from the incorporation of mosambi peel (Younis et al., 2019).

The carotenoid content and antioxidant action of citrus fruit fiber altered the brightness of bologna sausages throughout a wide concentration range (0.5%, 1%, 1.5%, and 2%). This included changes in the intensity of the red and yellow colors (Fernandez-Gines et al., 2003). Irradiated hamburgers (2 and 4 kGy) retained their red color (a*) when dried tomato peel (3% to 6%) was added to the meat (Luisa García, Calvo & Dolores Selgas, 2009). Sausage with 0.6%, 0.9%, or 1.2% dehydrated tomato peel kept its red hue for 21 days, but the brightness was dimmed and the yellow intensity (b*) was boosted, and there was an improvement in hardness and cutting force, but a decline in cohesiveness (Calvo, García & Selgas, 2008). Here, tomato peel’s high lycopene and beta-carotene concentration proved superior to pulp’s in preserving the red color of final meat products (Joseph et al., 2014). A few other examples showed the changes in different characteristics of meat products by incorporating various plant-based materials, especially waste, and have been listed in Table 1.

### Effect of dietary fiber incorporation on shelf-life of meat products

It is of the utmost importance that the quality and shelf stability of a meat product is maintained during its storage. It has been discovered that the addition of different kinds of fiber sources to meat products can alter the preservation quality in a variety of ways. The inclusion of chia seeds in (camburger) camel burger, too has recorded a reduced TBA (thiobarbituric acid) value, which implies lesser lipid oxidation in comparison to the control, and these burgers were determined to be organoleptically acceptable after storage for a period of 12 days (Zaki, 2018). Because it better inhibits oxymyoglobin oxidation, the addition of oat flour to chicken kofta has resulted in a product that is microbiologically safe and sensorial acceptable over the entire period of 15 days of storage (Lin & Lin, 2006). Grape seed, which includes flavonoids including catechin, epicatechin, procyanidins, and other chemicals with antimicrobial properties, is the most widely utilized and documented industrial waste for this purpose (Friedman, 2014). Uncertain processes, including disruption of the cytoplasmic membrane, blockage of specific metabolic pathways and enzymes, and chelation of critical metals for growth like zinc and iron, underlie the antibacterial effects of polyphenols and other natural chemicals (Daglia, 2012). Incorporation of grape seed in raw pork in aerobic packaging at 20 °C, when inoculated with 10^5^ CFU of *Listeria monocytogenes*, *Staphylococcus aureus*, and *Salmonella enterica*. Decreased growth of L. *monocytogenes* by 17.5%, *S*. aureus by 14%, and *S*. entericaby 20% (Shan et al., 2009). A 4-log decrease in the growth of L. *monocytogenes* at 4 °C, 1 log at 7 °C, and no changes at 12 °C was noticed in meat paté incorporated with pomegranate peel. The inoculation was done with 4 log CFU/g of L. *monocytogenes* at 4 °C, 7 °C, and 12 °C for 46 days (Hayrapetyan, Hazeleger & Beumer, 2012). A few other examples have been listed in Table 2, indicating the shelf-life through changes in microbiological characteristics and antioxidant capacity of different meat products as influenced by various incorporations.

**Table 2:** Indication of shelf-life through changes in microbiological characteristics and antioxidant capacity of different meat products as influenced by various incorporations.

Product	Incorporation	Characteristics before incorporation at 0 day of study	Characteristics after incorporation at 0 day of study	References
Pork chorizo	Oregano essential oil (0.1%)	Antioxidant capacity (DPPH)–26.48% Mesophilic aerobes–4.19 log cfu/g	Antioxidant capacity (DPPH)–27.42% Mesophilic aerobes–4.19 log cfu/g	Perales-Jasso et al. (2018)
Beef patty	Blueberry pomace extract (50 g/500ml)	TBA number–0.619 Protein carbonyl content–1.219 nmol carbonyl/mg protein Coliform bacteria–3.62 log cfu/g	TBA number–0.390 Protein carbonyl content–0.463 nmol carbonyl/mg protein Coliform bacteria–3.50 log cfu/g	Babaoğlu et al. (2022)
Beef patty	Blackberry pomace extract (50 g/500 ml)	TBA number–0.619 Protein carbonyl content–1.219 nmol carbonyl/mg protein Coliform bacteria–3.62 log cfu/g	TBA number–0.355 Protein carbonyl content–0.621 nmol carbonyl/mg protein Coliform bacteria–3.60 log cfu/g	Babaoğlu et al. (2022)
Beef sausage	White cauliflower leaf midribs powder (7.5%)	TBA value–0.234 mg/kg sample TVB–N–4.88 mg/kg sample	TBA value–0.132 mg/kg sample TVB–N–5.72 mg/kg sample	Abul-Fadl (2012)
Sausage	Pomegranate peel (3%)	TBA–0.237 mg of malonaldhyde/kg TVN–8.38 mg nitrogen/100 g sample Yeast and mold count–2 log cfu/g Coliform count–1.86 log cfu/g	TBA–0.235 mg of malonaldhyde/kg TVN–8.00 mg nitrogen/100 g sample Yeast and mold count–2.8 log cfu/g Coliform count–2 log cfu/g	El-Nashi et al. (2015)

### Health benefits by interaction of dietary fiber in meat products

Fortifying processed meat products with bioactive components that may help or mitigate the negative health consequences of eating processed meat is one strategy for combating the potential detrimental effects of eating processed meat. Studies reported a correlation between eating meat products incorporated with dietary fiber improves digestive health system, improve digestion, reduces the risk of coronary heart diseases and many more. Fortifying pork sausages with inulin from chicory root has been demonstrated to have considerable effects on the metabolites created in the gastrointestinal tract by the gut microbiota, as shown in a recent study using a rat model (Thøgersen et al., 2018). The short-chain fatty acids acetate, propionate, and butyrate have been identified as pivotal in the positive effects linked with dietary fiber consumption (Zhao et al., 2018; Gill et al., 2018), and their production was increased by fortification of pork sausage with inulin. Butyrate production was also increased by the inclusion of dietary fiber in salami (a fermented meat product) in a human intervention study conducted by Pérez-Burillo et al. (2020). Additionally, dietary butyrylated starch has showed to improve gut short-chain fatty acid content and reduce the production of harmful and carcinogenic O6-methyl-2-deoxyguanosine adducts, the latter of which has been linked to a diet high in red meat (Le Leu et al., 2015). Therefore, recent data suggests that compounds containing fermentable dietary fiber and short-chain fatty acids can mitigate the negative effects of eating processed meat products in the colon. While research into the cancer-fighting potential of unfermentable fiber is still limited, preliminary results from animal model studies are promising in cancer prevention (Corpet & Pierre, 2003).

Colon health benefits from high calcium consumption have been suggested by cohort studies (Huncharek, Muscat & Kupelnick, 2009; Meng et al., 2019). Recent research by Thøgersen et al., (2018) in a rat model examined the impact of supplementing processed meat with calcium and inulin. Interestingly, consuming processed meat that has been fortified with calcium-rich milk minerals decreases the formation of undesirable N-nitroso compounds in the gastrointestinal tract and increases the formation of short-chain fatty acids in the colon, compared to eating processed meat that has not been fortified (Thøgersen et al., 2018). In light of these encouraging findings, it appears that potentially harmful effects associated with the consumption of meat can, in fact, be mitigated through the modification of the meat product matrix and the fortification of meat products or through the strategic design of meals that include components like dietary fiber and calcium which counteract unintended effects in the intestinal system that are associated with the consumption of meat.

Once it was discovered that obesity was linked to mortality-inducing conditions like cardiovascular disease, it became clear that this issue needed urgent attention. In meat product formulations, particularly emulsified products that are known to have a high energy value, the addition of dietary fiber can result in a reduction in total energy consumption (Blackwood et al., 2016). This is because dietary fiber acts as fat replacers, in addition to delaying gastric emptying and increasing the distension of the stomach, both of which contribute to a greater perception of satiety (Hoad et al., 2004). Perception of satiety avoids the intake of excess calories and helps in reducing the obesity. In a research, appetite study and *in-vitro* digestibility of meat product (bologna sausage) incorporated with chia mucilage was conducted (Câmara et al., 2020). It was found that chia mucilage was proven as effective fat replacer in bologna sausage with increased retention time in stomach and consequently resulted in same satiety (Câmara et al., 2020). This will prevent excess eating, and one may avoid obesity by consuming such soluble fiber-based formulations of meat products.

In another study, a meat product (sausage) was formulated using cereal (rice) flour. It was claimed that a daily serving size of 100 g of sausage may correct a potassium and iron deficiency, nearly restore magnesium, calcium, and vitamin A, and cut a dietary fiber deficiency in half (Sadovoy et al., 2021). Also, laboratory mice whose staple diet included the formed sausage showed a significantly increased biological value and safety, as measured by cytological examinations of their blood (Sadovoy et al., 2021). Thus, meat products with dietary fiber from various fruits and vegetables may have health benefits for several disorders and chronic diseases. Eating meat products enriched with dietary fiber and plant-based materials may remove multiple deficiencies.

## Conclusion

A sustainable food system aims to deliver food security and nutrition for all, keeping future generations in view. In recent years it has been noted that almost one-third of the total food produced is lost or wasted worldwide during food production or in the food processing system. Moreover, utilization of these food waste materials can contribute to the alleviation of food insecurity. Since, these food waste materials possess important bioactive compounds and incorporation of these into meat products could enhance the nutritional and functional characteristics of newly developed meat product. In this review, we found that incorporating several fruit and vegetable wastes in their extract or powder form into processed meat products had a very significant enrichment. Incorporating these agro-industrial wastes enhances the products’ sensory and textural qualities and shelf-life, as they are a rich source of DF, several antioxidants, and health-promoting agents. Since, meat products contain a high percentage of fat especially saturated fat and devoid of DF, which poses several health problems like cardiovascular and gastrointestinal diseases. The health conscious consumer is becoming increasingly aware of the importance of balancing flavor and nutrition. Therefore, to overcome this problem, utilization of these food waste materials could pave the way to improve the food insecurity as well as functional attributes. In the future, the incorporation of transformed low-cost food waste-derived products or diet may provide the possibility to lower food costs and this could be a strong incentive for the stakeholders in the business industry to get involved in the utilization of food waste in meat processing industries given that quality and safety are guaranteed. Furthermore, since there are wide ranges of food waste materials around the globe and its scope of utilization, food communities are needed to carry such more investigation concerning the physicochemical characteristics to improve food sustainable system. Finally, food waste recycling in processed meat for human consumption may contribute to the reduction of the environmental impact, improve environmental footprints, and help meet the requirement of a sustainable food system.

## Additional Information and Declarations

### Data Availability

The following information was supplied regarding data availability: This is a literature review.

## References

[ref-1] Abul-Fadl MM (2012). Nutritional and chemical evaluation of white cauliflower by-products flour and the effect of its addition on beef sausage quality. Journal of Applied Sciences Research.

[ref-2] Ahmad S, Jafarzadeh S, Ariffin F, Zainul Abidin S (2020). Evaluation of physicochemical, antioxidant and antimicrobial properties of chicken sausage incorporated with different vegetables. Italian Journal of Food Science.

[ref-3] Aleson-Carbonell L, Fernandez-Lopez J, Sayas-Barbera E, Sendra E, Perez-Alvarez JA (2003). Utilization of lemon albedo in dry-cured sausages. Journal of Food Science.

[ref-4] Aleson-Carbonell L, Fernández-López J, Pérez-Alvarez JA, Kuri V (2005). Functional and sensory effects of fibre-rich ingredients on breakfast fresh sausages manufacture. Food Science and Technology International.

[ref-5] Amaral AB, Da Silva MV, Da Silva Lannes SCS (2018). Lipid oxidation in meat: mechanisms and protective factors—a review. Food Science and Technology.

[ref-6] Ani PN, Abel HC (2018). Nutrient, phytochemical, and antinutrient composition of *Citrus maxima* fruit juice and peel extract. Food Science & Nutrition.

[ref-7] Antonyak H, Iskra R, Panas N, Lysiuk R, Malavolta M, Mocchegiani E (2018). Selenium. Trace Elements and Minerals in Health and Longevity. Healthy Ageing and Longevity.

[ref-119] Babaoğlu AS, Unal K, Dilek NM, Poçan HB, Karakaya M (2022). Antioxidant and antimicrobial effects of blackberry, black chokeberry, blueberry, and red currant pomace extracts on beef patties subject to refrigerated storage. Meat Science.

[ref-8] Banerjee DK, Das AK, Banerjee R, Pateiro M, Nanda PK, Gadekar YP, Biswas S, McClements DJ, Lorenzo JM (2020). Application of Enoki Mushroom (*Flammulina Velutipes*) stem wastes as functional ingredients in goat meat nuggets. Foods.

[ref-9] Bhat Z (2011). Functional meat products: a review. International Journal of Meat Science.

[ref-10] Biesalski H-K (2005). Meat as a component of a healthy diet—are there any risks or benefits if meat is avoided in the diet?. Meat Science.

[ref-11] Blackwood AD, Salter J, Dettmar PW, Chaplin MF (2016). Dietary fibre, physicochemical properties and their relationship to health. Journal of the Royal Society for the Promotion of Health.

[ref-12] Calderón-Oliver M, López-Hernández LH (2022). Food vegetable and fruit waste used in meat products. Food Reviews International.

[ref-13] Calvo MM, García ML, Selgas MD (2008). Dry fermented sausages enriched with lycopene from tomato peel. Meat Science.

[ref-14] Câmara AKFI, Geraldi MV, Okuro PK, Maróstica MR, da Cunha RL, Pollonio MAR (2020). Satiety and in vitro digestibility of low saturated fat Bologna sausages added of chia mucilage powder and chia mucilage-based emulsion gel. Journal of Functional Foods.

[ref-15] Chan W (2004). Human nutrition | Macronutrients in meat. Encyclopedia of Meat Sciences.

[ref-16] Chappalwar AM, Pathak V, Goswami M, Verma AK (2020). Development of functional chicken patties with incorporation of mango peel powder as fat replacer. Nutrition & Food Science.

[ref-17] Choe JH, Kim HY, Kim CJ (2017). Effect of persimmon peel (Diospyros kaki Thumb.) extracts on lipid and protein oxidation of raw ground pork during refrigerated storage. Korean Journal for Food Science of Animal Resources.

[ref-18] Choe JH, Kim HY, Kim YJ, Yeo EJ, Kim CJ (2014). Antioxidant activity and phenolic content of persimmon peel extracted with different levels of ethanol. International Journal of Food Properties.

[ref-19] Choi YS, Choi JH, Han DJ, Kim HY, Lee MA, Kim HW, Jeong JY, Kim CJ (2011). Effects of rice bran fiber on heat-induced gel prepared with pork salt-soluble meat proteins in model system. Meat Science.

[ref-20] Christodoulides S, Dimidi E, Fragkos KC, Farmer AD, Whelan K, Scott SM (2016). Systematic review with meta-analysis: effect of fibre supplementation on chronic idiopathic constipation in adults. Alimentary Pharmacology & Therapeutics.

[ref-21] Colmenero FJ, Ayo MJ, Carballo J (2005). Physicochemical properties of low sodium frankfurter with added walnut: effect of transglutaminase combined with caseinate, KCl and dietary fibre as salt replacers. Meat Science.

[ref-22] Corpet DE, Pierre F (2003). Point: from animal models to prevention of colon cancer. Systematic review of chemoprevention in min mice and choice of the model system. Cancer Epidemiology, Biomarkers & Prevention: A Publication of the American Association for Cancer Research, Cosponsored by the American Society of Preventive Oncology.

[ref-23] Costabile G, Griffo E, Cipriano P, Vetrani C, Vitale M, Mamone G, Rivellese AA, Riccardi G, Giacco R (2018). Subjective satiety and plasma PYY concentration after wholemeal pasta. Appetite.

[ref-24] da Silva Frasao B, Lima Dos Santos Rosario AI, Leal Rodrigues B, Abreu Bitti H, Diogo Baltar J, Nogueira RI, Pereira da Costa M, Conte-Junior CA (2021). Impact of juçara (Euterpe edulis) fruit waste extracts on the quality of conventional and antibiotic-free broiler meat. Poultry Science.

[ref-25] Daglia M (2012). Polyphenols as antimicrobial agents. Current Opinion in Biotechnology.

[ref-116] Damodaran S, Parkin KL (2017). Fennema’s Food Chemistry, Fifth Edition. CRC Press.

[ref-26] Daniel CR, Cross AJ, Koebnick C, Sinha R (2011). Trends in meat consumption in the USA. Public Health Nutrition.

[ref-27] Devi MKA, Gondi M, Sakthivelu G, Giridhar P, Rajasekaran T, Ravishankar GA (2009). Functional attributes of soybean seeds and products, with reference to isoflavone content and antioxidant activity. Food Chemistry.

[ref-28] Díaz-Vela J, Totosaus A, Pérez-Chabela ML (2015). Integration of agroindustrial co-products as functional food ingredients: cactus pear (*O puntia ficus indica*) flour and pineapple (*A nanas comosus*) peel flour as fiber source in cooked sausages inoculated with lactic acid bacteria. Journal of Food Processing and Preservation.

[ref-29] Ekholm P, Virkki L, Ylinen M, Johansson L (2003). The effect of phytic acid and some natural chelating agents on the solubility of mineral elements in oat bran. Food Chemistry.

[ref-30] Elkhalifa AEO, Al-Shammari E, Adnan M, Alcantara JC, Mehmood K, Eltoum NE, Awadelkareem AM, Khan MA, Ashraf SA (2021). Development and characterization of novel biopolymer derived from *Abelmoschus esculentus* L. Extract and its antidiabetic potential. Molecules.

[ref-117] El-Nashi HB, Abdel Fattah AFAK, Abdel Rahman NR, Abd El-Razik MM (2015). Quality characteristics of beef sausage containing pomegranate peels during refrigerated storage. Annals of Agricultural Sciences.

[ref-31] Fallah A, Mohammad-Hasani A, Colagar AH (2018). Zinc is an essential element for male fertility: a review of Zn roles in men’s health, germination, sperm quality, and fertilization. Journal of Reproduction & Infertility.

[ref-32] Falowo AB, Fayemi PO, Muchenje V (2014). Natural antioxidants against lipid-protein oxidative deterioration in meat and meat products: a review. Food Research International.

[ref-33] Fernandez-Gines JM, Fernandez-Lopez J, Sayas-Barbera E, Sendra E, Perez-Alvarez JA (2003). Effect of storage conditions on quality characteristics of Bologna sausages made with citrus fiber. Journal of Food Science.

[ref-34] Flora SJS, Pachauri V (2010). Chelation in metal intoxication. International Journal of Environmental Research and Public Health.

[ref-35] Friedman M (2013). Anticarcinogenic, cardioprotective, and other health benefits of tomato compounds lycopene, α-tomatine, and tomatidine in pure form and in fresh and processed tomatoes. Journal of Agricultural and Food Chemistry.

[ref-36] Friedman M (2014). Antibacterial, antiviral, and antifungal properties of wines and winery byproducts in relation to their flavonoid content. Journal of Agricultural and Food Chemistry.

[ref-37] Future Market Insights Global and Consulting Pvt. Ltd (2022). Processed meat market by type, packaging, meat type & region—forecast 2022–2032. Delaware, United States.

[ref-38] Gibson GR, Probert HM, Van Loo J, Rastall RA, Roberfroid MB (2004). Dietary modulation of the human colonic microbiota: updating the concept of prebiotics. Nutrition Research Reviews.

[ref-39] Gill PA, van Zelm MC, Muir JG, Gibson PR (2018). Review article: short chain fatty acids as potential therapeutic agents in human gastrointestinal and inflammatory disorders. Alimentary Pharmacology & Therapeutics.

[ref-40] Giromini C, Givens DI (2022). Benefits and risks associated with meat consumption during key life processes and in relation to the risk of chronic diseases. Foods.

[ref-41] Gómez-Pinilla F (2008). Brain foods: the effects of nutrients on brain function. Nature Reviews Neuroscience.

[ref-115] Hariharan S, Dharmaraj S (2020). Selenium and selenoproteins: it’s role in regulation of inflammation. Inflammopharmacology.

[ref-42] Hayrapetyan H, Hazeleger WC, Beumer RR (2012). Inhibition of *Listeria monocytogenes* by pomegranate (*Punica granatum*) peel extract in meat paté at different temperatures. Food Control.

[ref-43] Hoad CL, Rayment P, Spiller RC, Marciani L, De Celis Alonso B, Traynor C, Mela DJ, Peters HPF, Gowland PA (2004). *In Vivo* imaging of intragastric gelation and its effect on satiety in humans. The Journal of Nutrition.

[ref-44] Hugo CJ, Hugo A (2015). Current trends in natural preservatives for fresh sausage products. Trends in Food Science & Technology.

[ref-45] Huncharek M, Muscat J, Kupelnick B (2009). Colorectal cancer risk and dietary intake of calcium, vitamin D, and dairy products: a meta-analysis of 26,335 cases from 60 observational studies. Nutrition and Cancer.

[ref-46] Jayadev A (2017). Comparative analysis of nutritional and anti nutritional components of selected citrus fruit species. International Journal for Research in Applied Science and Engineering Technology.

[ref-47] Jayasena DD, Jo C (2013). Essential oils as potential antimicrobial agents in meat and meat products: a review. Trends in Food Science & Technology.

[ref-48] Jiao J, Xu J-Y, Zhang W, Han S, Qin L-Q (2015). Effect of dietary fiber on circulating C-reactive protein in overweight and obese adults: a meta-analysis of randomized controlled trials. International Journal of Food Sciences and Nutrition.

[ref-49] Joseph S, Chatli MK, Biswas AK, Sahoo J (2014). Oxidative stability of pork emulsion containing tomato products and pink guava pulp during refrigerated aerobic storage. Journal of Food Science and Technology.

[ref-50] Kim HW, Setyabrata D, Lee Y, Jones OG, Kim YHB (2017). Effect of house cricket (*Acheta domesticus*) flour addition on physicochemical and textural properties of meat emulsion under various formulations. Journal of Food Science.

[ref-51] Latunde-Dada GO, Neale RJ (2007). Review: availability of iron from foods. International Journal of Food Science & Technology.

[ref-52] Lawrie RA, Ledward DA (2014). Lawrie’s Meat Science.

[ref-53] Le Leu RK, Winter JM, Christophersen CT, Young GP, Humphreys KJ, Hu Y, Gratz SW, Miller RB, Topping DL, Bird AR, Conlon MA (2015). Butyrylated starch intake can prevent red meat-induced O_6_-methyl-2-deoxyguanosine adducts in human rectal tissue: a randomised clinical trial. British Journal of Nutrition.

[ref-54] Lin Y, Chen K, Tu D, Yu X, Dai Z, Shen Q (2019). Characterization of dietary fiber from wheat bran (*Triticum aestivum* L.) and its effect on the digestion of surimi protein. LWT.

[ref-55] Lin KW, Lin HY (2006). Quality characteristics of Chinese-style meatball containing bacterial cellulose (Nata). Journal of Food Science.

[ref-56] Liu RH (2004). Potential synergy of phytochemicals in cancer prevention: mechanism of action. The Journal of Nutrition.

[ref-57] Liu X, Wu Y, Li F, Zhang D (2015). Dietary fiber intake reduces risk of inflammatory bowel disease: result from a meta-analysis. Nutrition Research.

[ref-58] Luisa García M, Calvo MM, Dolores Selgas M (2009). Beef hamburgers enriched in lycopene using dry tomato peel as an ingredient. Meat Science.

[ref-59] Luisa García M, Cáceres E, Dolores Selgas M (2006). Effect of inulin on the textural and sensory properties of mortadella, a Spanish cooked meat product. International Journal of Food Science and Technology.

[ref-60] Mamma D, Christakopoulos P (2014). Biotransformation of citrus by-products into value added products. Waste and Biomass Valorization.

[ref-61] McDowell LR (2008). Vitamins in animal and human nutrition.

[ref-62] McRae MP (2017). Dietary fiber is beneficial for the prevention of cardiovascular disease: an umbrella review of meta-analyses. Journal of Chiropractic Medicine.

[ref-63] Meng Y, Sun J, Yu J, Wang C, Su J (2019). Dietary intakes of calcium, iron, magnesium, and potassium elements and the risk of colorectal cancer: a meta-analysis. Biological Trace Element Research.

[ref-64] Mhurchu CN, Dunshea-Mooij C, Bennett D, Rodgers A (2005). Effect of chitosan on weight loss in overweight and obese individuals: a systematic review of randomized controlled trials. Obesity Reviews.

[ref-65] Micha R, Michas G, Mozaffarian D (2012). Unprocessed red and processed meats and risk of coronary artery disease and type 2 diabetes—an updated review of the evidence. Current Atherosclerosis Reports.

[ref-66] Montoya L, Quintero N, Ortiz S, Lopera J, Millán P, Rodríguez-Stouvenel A (2022). Inulin as a fat-reduction ingredient in pork and chicken meatballs: its effects on physicochemical characteristics and consumer perceptions. Foods.

[ref-67] Nweze CC, Nebechukwu EW, Bawa MY (2021). Dietary fiber and risk of coronary heart diseases. GSC Advanced Research and Reviews.

[ref-68] Obeid R, Heil SG, Verhoeven MMA, van den Heuvel EGHM, de Groot LCPGM, Eussen SJPM (2019). Vitamin B12 intake from animal foods, biomarkers, and health aspects. Frontiers in Nutrition.

[ref-69] Okarter N, Liu RH (2010). Health benefits of whole grain phytochemicals. Critical Reviews in Food Science and Nutrition.

[ref-118] Perales-Jasso YJ, Gamez-Noyola SA, Aranda-Ruiz J, Hernandez-Martinez CA, Gutierrez-Soto G, Luna-Maldonado AI, Silva-Vazquez R, Hume ME, Mendez-Zamora G (2018). Oregano powder substitution and shelf life in pork chorizo using Mexican oregano essential oil. Food Science & Nutrition.

[ref-73] Pérez-Burillo S, Pastoriza S, Gironés A, Avellaneda A, Pilar Francino M, Rufián-Henares JA (2020). Potential probiotic salami with dietary fiber modulates metabolism and gut microbiota in a human intervention study. Journal of Functional Foods.

[ref-70] Pins (2006). A review of the effects of barley beta-glucan on cardiovascular and diabetic risk. Cereal Foods World.

[ref-71] Pintado T, Herrero AM, Jiménez-Colmenero F, Ruiz-Capillas C (2016). Strategies for incorporation of chia (*Salvia hispanica* L.) in frankfurters as a health-promoting ingredient. Meat Science.

[ref-72] Puupponen-Pimiä R, Aura A-M, Oksman-Caldentey KM, Myllärinen P, Saarela M, Mattila-Sandholm T, Poutanen K (2002). Development of functional ingredients for gut health. Trends in Food Science & Technology.

[ref-74] Quezada N, Cherian G (2012). Lipid characterization and antioxidant status of the seeds and meals of *Camelina sativa* and flax. European Journal of Lipid Science and Technology.

[ref-75] Raman M, Nilsson U, Skog K, Lawther M, Nair B, Nyman M (2013). Physicochemical characterisation of dietary fibre components and their ability to bind some process-induced mutagenic heterocyclic amines, Trp-P-1, Trp-P-2, AαC and MeAαC. Food Chemistry.

[ref-76] Rashwan AK, Karim N, Shishir MRI, Bao T, Lu Y, Chen W (2020). Jujube fruit: a potential nutritious fruit for the development of functional food products. Journal of Functional Foods.

[ref-77] Ritchie H, Rosado P, Roser M (2017). Meat and dairy production. Our World in Data.

[ref-78] Sadovoy V, Shchedrina T, Trubina I, Morgunova A, Franko E (2021). Cooked sausage enriched with essential nutrients for the gastrointestinal diet. Foods and Raw Materials.

[ref-79] Salejda AM, Olender K, Zielińska-Dawidziak M, Mazur M, Szperlik J, Miedzianka J, Zawiślak I, Kolniak-Ostek J, Szmaja A (2022). Frankfurter-type sausage enriched with buckwheat by-product as a source of bioactive compounds. Foods.

[ref-80] Sánchez-Ortega I, García-Almendárez BE, Santos-López EM, Amaro-Reyes A, Barboza-Corona JE, Regalado C (2014). Antimicrobial edible films and coatings for meat and meat products preservation. The Scientific World Journal.

[ref-81] Sánchez-Zapata E, Fernández-López J, Peñaranda M, Fuentes-Zaragoza E, Sendra E, Sayas E, Pérez-Alvarez JA (2011). Technological properties of date paste obtained from date by-products and its effect on the quality of a cooked meat product. Food Research International.

[ref-82] Sante-Lhoutellier V, Aubry L, Gatellier P (2007). Effect of oxidation on *in vitro* digestibility of skeletal muscle myofibrillar proteins. Journal of Agricultural and Food Chemistry.

[ref-83] Santhi D, Kalaikannan A, Natarajan A (2020). Characteristics and composition of emulsion-based functional low-fat chicken meat balls fortified with dietary fiber sources. Journal of Food Process Engineering.

[ref-84] Savadkoohi S, Hoogenkamp H, Shamsi K, Farahnaky A (2014). Color, sensory and textural attributes of beef frankfurter, beef ham and meat-free sausage containing tomato pomace. Meat Science.

[ref-85] Serdaroğlu M, Kavuşan HS, İpek G, Öztürk B (2018). Evaluation of the quality of beef patties formulated with dried pumpkin pulp and seed. Korean Journal for Food Science of Animal Resources.

[ref-86] Shan B, Cai YZ, Brooks JD, Corke H (2009). Antibacterial and antioxidant effects of five spice and herb extracts as natural preservatives of raw pork. Journal of the Science of Food and Agriculture.

[ref-87] Shan LC, De Brún A, Henchion M, Li C, Murrin C, Wall PG, Monahan FJ (2017). Consumer evaluations of processed meat products reformulated to be healthier—a conjoint analysis study. Meat Science.

[ref-88] Sharma S, Sheehy T, Kolonel LN (2013). Contribution of meat to vitamin B_12_, iron and zinc intakes in five ethnic groups in the USA: implications for developing food-based dietary guidelines. Journal of Human Nutrition and Dietetics: The Official Journal of the British Dietetic Association.

[ref-89] Sharma A, Yadav B, Ritika BY (2008). Resistant starch: physiological roles and food applications. Food Reviews International.

[ref-90] Shay J, Elbaz HA, Lee I, Zielske SP, Malek MH, Hüttemann M (2015). Molecular mechanisms and therapeutic effects of (−)-Epicatechin and other polyphenols in cancer, inflammation, diabetes, and neurodegeneration. Oxidative Medicine and Cellular Longevity.

[ref-91] Slavin J (2013). Fiber and prebiotics: mechanisms and health benefits. Nutrients.

[ref-92] Soliman GA (2019). Dietary fiber, atherosclerosis, and cardiovascular disease. Nutrients.

[ref-94] Stephen AM, Champ MMJ, Cloran SJ, Fleith M, van Lieshout L, Mejborn H, Burley VJ (2017). Dietary fibre in Europe: current state of knowledge on definitions, sources, recommendations, intakes and relationships to health. Nutrition Research Reviews.

[ref-95] Stevenson L, Phillips F, O’sullivan K, Walton J (2012). Wheat bran: its composition and benefits to health, a European perspective. International Journal of Food Sciences and Nutrition.

[ref-96] Sun W, Zhou F, Zhao M (2015). Cantonese sausage, processing, storage and composition. Processing and Impact on Active Components in Food.

[ref-97] Thøgersen R, Castro-Mejía JL, Sundekilde UK, Hansen LH, Hansen AK, Nielsen DS, Bertram HC (2018). Ingestion of an inulin-enriched pork sausage product positively modulates the gut microbiome and metabolome of healthy rats. Molecular Nutrition & Food Research.

[ref-98] Thompson SV, Hannon BA, An R, Holscher HD (2017). Effects of isolated soluble fiber supplementation on body weight, glycemia, and insulinemia in adults with overweight and obesity: a systematic review and meta-analysis of randomized controlled trials. The American Journal of Clinical Nutrition.

[ref-99] Ur Rahman U, Sahar A, Ishaq A, Aadil RM, Zahoor T, Ahmad MH (2018). Advanced meat preservation methods: a mini review. Journal of Food Safety.

[ref-100] USDA (2005). US department of health and human services. Dietary guidelines for Americans. https://health.gov/dietaryguidelines/2005.asp.

[ref-101] Valsta LM, Tapanainen H, Männistö S (2005). Meat fats in nutrition. Meat Science.

[ref-102] Vural Z, Avery A, Kalogiros DI, Coneyworth LJ, Welham SJM (2020). Trace mineral intake and deficiencies in older adults living in the community and institutions: a systematic review. Nutrients.

[ref-103] Wichert B, Schuster S, Hofmann M, Dobenecker B, Kienzle E (2002). Influence of different cellulose types on feces quality of dogs. The Journal of Nutrition.

[ref-104] Xiong RG, Zhou DD, Wu SX, Huang SY, Saimaiti A, Yang ZJ, Shang A, Zhao CN, Gan RY, Li HB (2022). Health benefits and side effects of short-chain fatty acids. Foods.

[ref-105] Yang J, Wang HP, Zhou L, Xu CF (2012). Effect of dietary fiber on constipation: a meta analysis. World Journal of Gastroenterology.

[ref-106] Yeung CK, Huang SC (2017). Effects of food proteins on sensory and physico-chemical properties of emulsified pork meatballs. Journal of Food and Nutrition Research.

[ref-107] Younis K, Ahmad S, Malik MA (2021). Mosambi peel powder incorporation in meat products: effect on physicochemical properties and shelf life stability. Applied Food Research.

[ref-108] Younis K, Ahmad S, Osama K, Malik MA (2019). Optimization of de-bittering process of mosambi (*Citrus limetta*) peel: artificial neural network, Gaussian process regression and support vector machine modeling approach. Journal of Food Process Engineering.

[ref-109] Zając M, Guzik P, Kulawik P, Tkaczewska J, Florkiewicz A, Migdał W (2019). The quality of pork loaves with the addition of hemp seeds, de-hulled hemp seeds, hemp protein and hemp flour. LWT.

[ref-110] Zaki E (2018). Impact of adding chia seeds (Salvia hispanica) on the quality properties of camel burger “Camburger” during cold storage. International Journal of Current Microbiology and Applied Sciences.

[ref-111] Zhao L, Zhang F, Ding X, Wu G, Lam YY, Wang X, Fu H, Xue X, Lu C, Ma J, Yu L, Xu C, Ren Z, Xu Y, Xu S, Shen H, Zhu X, Shi Y, Shen Q, Dong W, Liu R, Ling Y, Zeng Y, Wang X, Zhang Q, Wang J, Wang L, Wu Y, Zeng B, Wei H, Zhang M, Peng Y, Zhang C (2018). Gut bacteria selectively promoted by dietary fibers alleviate type 2 diabetes. Science.

